# 4-(4-Chloro­phen­yl)-5-phenyl­isoxazole

**DOI:** 10.1107/S1600536809034254

**Published:** 2009-09-05

**Authors:** M. Krishnaiah, R. Ravi Kumar, Thanzaw Oo, Pho Kaung

**Affiliations:** a Department of Physics, SV University, Tirupati 517502, India; b Department of Physics, Yangon University, Myanmar

## Abstract

The title compound, C_15_H_10_ClNO, is a functionalized isoxazole with a chloro­phenyl and a phenyl substitutent. The mean plane of the isoxazole ring is inclined to those of the two benzene ring mean planes by 38.32 (16) and 43.91 (18)°.

## Related literature

For the chemistry and biological properties of isoxazoles, see: Bruno *et al.* (2004 ▶); Foti *et al.* (2004 ▶); He *et al.* (2000 ▶); Lakhvich *et al.* (1989 ▶); Lin *et al.* (1997 ▶); Makarov *et al.* (2005 ▶); Shipman (1995 ▶); Zhong *et al.* (2005 ▶). For related structures, see: Chang (2007 ▶); Tang *et al.* (2006 ▶); Zhang *et al.* (2006 ▶). For the synthesis, see: Subba Raju & Rao (1987 ▶). 
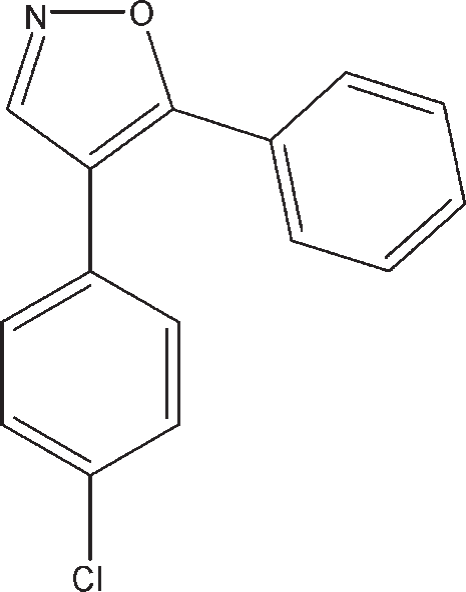


## Experimental

### 

#### Crystal data

C_15_H_10_ClNO
*M* *_r_* = 255.69
Monoclinic, 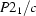

*a* = 6.554 (2) Å
*b* = 25.966 (2) Å
*c* = 7.4721 (19) Å
β = 106.171 (3)°
*V* = 1221.2 (5) Å^3^
*Z* = 4
Mo *K*α radiation
μ = 0.30 mm^−1^
*T* = 295 K
0.3 × 0.2 × 0.2 mm

#### Data collection

Bruker SMART CCD diffractometer
Absorption correction: multi-scan (*SADABS*; Bruker, 2001 ▶) *T*_min_ = 0.928, *T*_max_ = 0.952
2820 measured reflections
2132 independent reflections
1851 reflections with *I* > 2σ(*I*)
*R*_int_ = 0.050

#### Refinement

*R*[*F* ^2^ > 2σ(*F* ^2^)] = 0.063
*wR*(*F* ^2^) = 0.197
*S* = 1.15
2132 reflections
204 parameters
All H-atom parameters refined
Δρ_max_ = 0.27 e Å^−3^
Δρ_min_ = −0.40 e Å^−3^

### 

Data collection: *SMART* (Bruker 2007 ▶); cell refinement: *SAINT* (Bruker 2007 ▶); data reduction: *SAINT*; program(s) used to solve structure: *SHELXS97* (Sheldrick, 2008 ▶); program(s) used to refine structure: *SHELXL97* (Sheldrick, 2008 ▶); molecular graphics: *ORTEP-3 for Windows* (Farrugia, 1997 ▶); software used to prepare material for publication: *enCIFer* (Allen *et al.*, 2004 ▶) and *PARST* (Nardelli, 1995 ▶).

## Supplementary Material

Crystal structure: contains datablocks I, global. DOI: 10.1107/S1600536809034254/su2135sup1.cif

Structure factors: contains datablocks I. DOI: 10.1107/S1600536809034254/su2135Isup2.hkl

Additional supplementary materials:  crystallographic information; 3D view; checkCIF report

## Figures and Tables

**Fig. 1.**
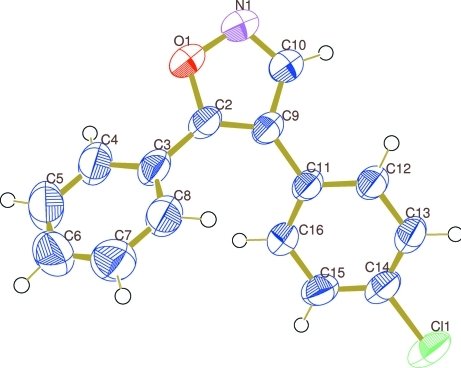
View of the molecular structure of the title compound, showing the atom-labelling scheme and displacement ellipsoids drawn at the 50% probability level.

## supplementary crystallographic information

### Comment

Isoxazoles are often used as pharmacophores in medicinal chemistry (Makarov *et al.*, 2005). They are also important intermediates in the synthesis of many complex natural products (Lakhvich *et al.*, 1989; Shipman, 1995). Recent synthetic efforts have established the importance of biologically active heterocyclic compounds (Foti *et al.*, 2004). Of particular importance are the derivatives of isoxazoles representing one of the most active classes of compounds widely used in agrochemicals and pharmaceuticals (He *et al.*, 2000). Such compounds have been studied from a synthetic (Bruno *et al.*, 2004) and also from a structural viewpoint (Zhong *et al.*, 2005). Isoxazole derivatives exhibit anticonvulsant, antibacterial, antiasthmatic, and other pharmacological activities (Lin *et al.*, 1997). In this article, we report on the crystal structure of the title compound, 4-(4-chloro phenyl)-5-phenylisoxazole.

The molecular structute of the title compound is illustrated in Fig. 1 and the geometrical parameters are avilable in the archived CIF. The title compound is a functionalized isoxazole with a chlorophenyl and a phenyl substituent at positions 4 (C9) and 5 (C2), respectively, on the five-membered heterocyclic ring. They are inclined to the planar isoxazole ring mean plane by 38.32 (16)° and 43.91 (18)°, respectively. The torsion angles [C10-C9-C11-C12 = 38.4 (4)°, C2-C9-C11-C16 = 36.6 (5)°, O1-C2-C3-C4 = 44.1 (4)°, and C9-C2-C3-C8 = 43.7 (5)°)] confirm that these rings are twisted with respect to the plane of the isoxazole ring. The bond lengths of the isoxazole ring are normal and comparable to those reported for related structures: [3-(4-Chlorophenyl)isoxazol-5-yl]methanol (Tang *et al.*, 2006), 3-(4-Chlorophenyl)isoxazole-5-carbaldehyde (Zhang *et al.*, 2006), and 3-(2-Chlorophenyl)-*N*-methylisoxazole-5-carboxamide (Chang, 2007). However, bond length C2-C9 [1.359 (4) A°] is lengthened compared to the corresponding values in the above three related structures. {1.337 (3), 1.334 (3), 1.336 (3) Å, respectively}. This may be due to the steric effects caused by the substituents attached at atoms C2 and C9 on the isoxazole ring.

### Experimental

The title compound was prepare according the the published procedure (Subba Raju & Rao, 1987). Recrystallization from n-hexane-benzene (1:1, v:v) by slow evaporation gave colourless block-like crystals suitable for X-ray diffraction analysis.

### Refinement

All the H-atoms were clearly located in difference electron-density maps and were freely refined: C-H = 0.91 (5) - 1.00 (4) Å.

### Crystal data

**Table:**

C_15_H_10_ClNO	*F*(000) = 528
*M**_r_* = 255.69	*D*_x_ = 1.391 Mg m^−^^3^*D*_m_ = 1.39 Mg m^−^^3^*D*_m_ measured by none
Monoclinic, *P*2_1_/*c*	Mo *K*α radiation, λ = 0.71069 Å
Hall symbol: -P 2ybc	Cell parameters from 2895 reflections
*a* = 6.554 (2) Å	θ = 2.4–25.0°
*b* = 25.966 (2) Å	µ = 0.30 mm^−^^1^
*c* = 7.4721 (19) Å	*T* = 295 K
β = 106.171 (3)°	Block, colourless
*V* = 1221.2 (5) Å^3^	0.3 × 0.2 × 0.2 mm
*Z* = 4	

### Data collection

**Table:**

Bruker CCD diffractometer	2132 independent reflections
Radiation source: fine-focus sealed tube	1851 reflections with *I* > 2σ(*I*)
graphite	*R*_int_ = 0.050
ω scans	θ_max_ = 25.0°, θ_min_ = 2.9°
Absorption correction: multi-scan (*SADABS*; Bruker, 2001)	*h* = −1→7
*T*_min_ = 0.928, *T*_max_ = 0.952	*k* = −1→30
2820 measured reflections	*l* = −8→8

### Refinement

**Table:**

Refinement on *F*^2^	Secondary atom site location: difference Fourier map
Least-squares matrix: full	Hydrogen site location: inferred from neighbouring sites
*R*[*F*^2^ > 2σ(*F*^2^)] = 0.063	All H-atom parameters refined
*wR*(*F*^2^) = 0.197	*w* = 1/[σ^2^(*F*_o_^2^) + (0.1057*P*)^2^ + 0.4763*P*] where *P* = (*F*_o_^2^ + 2*F*_c_^2^)/3
*S* = 1.15	(Δ/σ)_max_ = 0.013
2132 reflections	Δρ_max_ = 0.27 e Å^−^^3^
204 parameters	Δρ_min_ = −0.40 e Å^−^^3^
0 restraints	Extinction correction: *SHELXL97* (Sheldrick, 2008), Fc^*^=kFc[1+0.001xFc^2^λ^3^/sin(2θ)]^-1/4^
Primary atom site location: structure-invariant direct methods	Extinction coefficient: 0.024 (6)

### Special details

**Table:**

Geometry. All e.s.d.'s (except the e.s.d. in the dihedral angle between two l.s. planes) are estimated using the full covariance matrix. The cell e.s.d.'s are taken into account individually in the estimation of e.s.d.'s in distances, angles and torsion angles; correlations between e.s.d.'s in cell parameters are only used when they are defined by crystal symmetry. An approximate (isotropic) treatment of cell e.s.d.'s is used for estimating e.s.d.'s involving l.s. planes.
Refinement. Refinement of *F*^2^ against ALL reflections. The weighted *R*-factor *wR* and goodness of fit *S* are based on *F*^2^, conventional *R*-factors *R* are based on *F*, with *F* set to zero for negative *F*^2^. The threshold expression of *F*^2^ > σ(*F*^2^) is used only for calculating *R*-factors(gt) *etc*. and is not relevant to the choice of reflections for refinement. *R*-factors based on *F*^2^ are statistically about twice as large as those based on *F*, and *R*- factors based on ALL data will be even larger.

### Fractional atomic coordinates and isotropic or equivalent isotropic displacement parameters (Å^2^)

**Table:**

	*x*	*y*	*z*	*U*_iso_*/*U*_eq_	
N1	0.1131 (4)	0.71167 (11)	0.9809 (4)	0.0706 (8)	
O1	0.0578 (3)	0.65915 (8)	0.9619 (3)	0.0655 (6)	
C2	0.1585 (4)	0.63669 (11)	0.8469 (4)	0.0539 (7)	
C3	0.1165 (5)	0.58171 (11)	0.8114 (4)	0.0592 (7)	
C4	−0.0886 (6)	0.56265 (14)	0.7745 (5)	0.0757 (9)	
C5	−0.1270 (7)	0.51065 (16)	0.7433 (7)	0.0921 (12)	
C6	0.0349 (8)	0.47763 (15)	0.7496 (6)	0.0913 (12)	
C7	0.2396 (7)	0.49578 (15)	0.7837 (6)	0.0865 (11)	
C8	0.2801 (6)	0.54770 (13)	0.8143 (5)	0.0710 (9)	
C9	0.2800 (4)	0.67225 (11)	0.7910 (4)	0.0524 (7)	
C10	0.2441 (5)	0.71820 (12)	0.8794 (4)	0.0632 (8)	
C11	0.4076 (4)	0.66740 (10)	0.6569 (4)	0.0506 (6)	
C12	0.5940 (4)	0.69549 (11)	0.6830 (4)	0.0560 (7)	
C13	0.7069 (4)	0.69393 (11)	0.5508 (4)	0.0587 (7)	
C14	0.6323 (5)	0.66396 (11)	0.3947 (5)	0.0596 (8)	
Cl1	0.77032 (15)	0.66286 (4)	0.22763 (14)	0.0817 (4)	
C15	0.4495 (5)	0.63545 (13)	0.3666 (5)	0.0641 (8)	
C16	0.3369 (5)	0.63760 (11)	0.4988 (4)	0.0586 (7)	
H8	0.431 (7)	0.5603 (15)	0.846 (5)	0.086 (11)*	
H10	0.297 (5)	0.7525 (13)	0.865 (5)	0.069 (9)*	
H12	0.650 (5)	0.7156 (13)	0.801 (5)	0.073 (10)*	
H13	0.836 (6)	0.7127 (13)	0.568 (5)	0.070 (9)*	
H16	0.204 (6)	0.6189 (14)	0.476 (5)	0.075 (9)*	
H15	0.394 (6)	0.6146 (16)	0.256 (6)	0.094 (12)*	
H7	0.353 (7)	0.4749 (18)	0.780 (6)	0.102 (13)*	
H4	−0.208 (6)	0.5869 (15)	0.763 (5)	0.085 (11)*	
H6	0.014 (7)	0.4435 (19)	0.723 (6)	0.102 (13)*	
H5	−0.270 (8)	0.4997 (19)	0.722 (7)	0.122 (17)*	

### Atomic displacement parameters (Å^2^)

**Table:**

	*U*^11^	*U*^22^	*U*^33^	*U*^12^	*U*^13^	*U*^23^
N1	0.0793 (17)	0.0725 (17)	0.0737 (17)	−0.0026 (12)	0.0440 (14)	−0.0121 (12)
O1	0.0685 (13)	0.0744 (13)	0.0670 (13)	−0.0023 (10)	0.0411 (10)	−0.0038 (10)
C2	0.0534 (14)	0.0615 (16)	0.0551 (15)	0.0020 (11)	0.0289 (11)	0.0019 (12)
C3	0.0649 (16)	0.0626 (16)	0.0607 (17)	−0.0020 (12)	0.0351 (13)	0.0094 (12)
C4	0.0681 (19)	0.074 (2)	0.093 (3)	−0.0085 (16)	0.0371 (17)	0.0076 (18)
C5	0.085 (2)	0.081 (2)	0.115 (3)	−0.022 (2)	0.036 (2)	0.007 (2)
C6	0.119 (3)	0.062 (2)	0.097 (3)	−0.013 (2)	0.038 (2)	0.0002 (19)
C7	0.100 (3)	0.067 (2)	0.102 (3)	0.0114 (19)	0.044 (2)	0.0039 (18)
C8	0.0680 (19)	0.0662 (19)	0.088 (2)	0.0002 (15)	0.0364 (16)	0.0052 (16)
C9	0.0507 (14)	0.0587 (15)	0.0550 (16)	−0.0009 (11)	0.0267 (11)	0.0006 (11)
C10	0.0679 (17)	0.0631 (17)	0.0684 (19)	−0.0038 (13)	0.0349 (14)	−0.0058 (14)
C11	0.0521 (14)	0.0517 (14)	0.0565 (16)	0.0007 (10)	0.0291 (12)	0.0026 (11)
C12	0.0555 (15)	0.0555 (14)	0.0653 (18)	−0.0009 (11)	0.0307 (13)	0.0018 (13)
C13	0.0517 (15)	0.0607 (16)	0.0726 (19)	0.0001 (12)	0.0322 (13)	0.0093 (14)
C14	0.0620 (16)	0.0608 (16)	0.069 (2)	0.0128 (12)	0.0403 (14)	0.0125 (13)
Cl1	0.0872 (7)	0.0946 (7)	0.0854 (7)	0.0188 (4)	0.0606 (5)	0.0153 (4)
C15	0.0730 (19)	0.0686 (18)	0.0605 (18)	0.0039 (14)	0.0348 (14)	−0.0036 (14)
C16	0.0591 (16)	0.0638 (17)	0.0609 (17)	−0.0087 (13)	0.0300 (13)	−0.0042 (13)

### Geometric parameters (Å, °)

**Table:**

N1—C10	1.306 (4)	C8—H8	1.00 (4)
N1—O1	1.408 (3)	C9—C10	1.415 (4)
O1—C2	1.353 (3)	C9—C11	1.478 (4)
C2—C9	1.359 (4)	C10—H10	0.97 (3)
C2—C3	1.464 (4)	C11—C16	1.380 (4)
C3—C8	1.385 (4)	C11—C12	1.389 (4)
C3—C4	1.386 (5)	C12—C13	1.390 (4)
C4—C5	1.381 (6)	C12—H12	1.00 (4)
C4—H4	0.99 (4)	C13—C14	1.374 (5)
C5—C6	1.355 (6)	C13—H13	0.96 (4)
C5—H5	0.95 (5)	C14—C15	1.374 (5)
C6—C7	1.377 (6)	C14—Cl1	1.734 (3)
C6—H6	0.91 (5)	C15—C16	1.389 (4)
C7—C8	1.381 (5)	C15—H15	0.97 (4)
C7—H7	0.92 (5)	C16—H16	0.97 (4)
			
C10—N1—O1	105.0 (2)	C2—C9—C11	129.8 (3)
C2—O1—N1	108.9 (2)	C10—C9—C11	125.9 (2)
O1—C2—C9	109.5 (2)	N1—C10—C9	112.5 (3)
O1—C2—C3	115.8 (2)	N1—C10—H10	120 (2)
C9—C2—C3	134.8 (3)	C9—C10—H10	128 (2)
C8—C3—C4	118.6 (3)	C16—C11—C12	119.2 (3)
C8—C3—C2	120.9 (3)	C16—C11—C9	120.6 (2)
C4—C3—C2	120.5 (3)	C12—C11—C9	120.1 (3)
C5—C4—C3	120.2 (4)	C11—C12—C13	120.4 (3)
C5—C4—H4	120 (2)	C11—C12—H12	119 (2)
C3—C4—H4	119 (2)	C13—C12—H12	120 (2)
C6—C5—C4	120.6 (4)	C14—C13—C12	119.1 (3)
C6—C5—H5	123 (3)	C14—C13—H13	120 (2)
C4—C5—H5	116 (3)	C12—C13—H13	121 (2)
C5—C6—C7	120.2 (4)	C13—C14—C15	121.6 (3)
C5—C6—H6	123 (3)	C13—C14—Cl1	119.1 (2)
C7—C6—H6	117 (3)	C15—C14—Cl1	119.3 (3)
C6—C7—C8	119.8 (4)	C14—C15—C16	118.9 (3)
C6—C7—H7	123 (3)	C14—C15—H15	122 (2)
C8—C7—H7	117 (3)	C16—C15—H15	119 (2)
C7—C8—C3	120.5 (3)	C11—C16—C15	120.9 (3)
C7—C8—H8	119 (2)	C11—C16—H16	120 (2)
C3—C8—H8	120 (2)	C15—C16—H16	119 (2)
C2—C9—C10	104.0 (2)		
			
C10—N1—O1—C2	−0.6 (3)	C3—C2—C9—C11	4.8 (6)
N1—O1—C2—C9	0.7 (3)	O1—N1—C10—C9	0.3 (4)
N1—O1—C2—C3	−179.6 (2)	C2—C9—C10—N1	0.1 (4)
O1—C2—C3—C8	−135.8 (3)	C11—C9—C10—N1	175.5 (3)
C9—C2—C3—C8	43.7 (5)	C2—C9—C11—C16	36.6 (5)
O1—C2—C3—C4	44.1 (4)	C10—C9—C11—C16	−137.5 (3)
C9—C2—C3—C4	−136.4 (4)	C2—C9—C11—C12	−147.5 (3)
C8—C3—C4—C5	0.7 (5)	C10—C9—C11—C12	38.4 (4)
C2—C3—C4—C5	−179.2 (4)	C16—C11—C12—C13	0.5 (4)
C3—C4—C5—C6	0.4 (7)	C9—C11—C12—C13	−175.4 (3)
C4—C5—C6—C7	−1.2 (7)	C11—C12—C13—C14	−0.4 (4)
C5—C6—C7—C8	1.0 (7)	C12—C13—C14—C15	−0.2 (4)
C6—C7—C8—C3	0.2 (6)	C12—C13—C14—Cl1	178.9 (2)
C4—C3—C8—C7	−1.0 (5)	C13—C14—C15—C16	0.7 (5)
C2—C3—C8—C7	179.0 (3)	Cl1—C14—C15—C16	−178.4 (2)
O1—C2—C9—C10	−0.5 (3)	C12—C11—C16—C15	0.0 (4)
C3—C2—C9—C10	179.9 (3)	C9—C11—C16—C15	175.9 (3)
O1—C2—C9—C11	−175.6 (3)	C14—C15—C16—C11	−0.6 (5)
